# The Cycle of Equitable Codesign’ Using Restorative Practice Principles to Address Epistemic Injustice and Advance Codesign in Mental Health

**DOI:** 10.1111/hex.70746

**Published:** 2026-06-26

**Authors:** Michelle Kehoe

**Affiliations:** ^1^ Occupational Therapy Department Monash University, Peninsula Campus, Moorooduc Hwy Melbourne Victoria Australia; ^2^ Bayside Health (Alfred Care Group) Child and Adolescent Mental Health, Nepean Hwy, Moorabbin Melbourne Victoria Australia

## Abstract

**Background:**

Co‐design has become a prominent approach in mental health research, policy and service development, aiming to centre lived experience within decision‐making processes. Despite its promise, co‐design practices often fall short, with reports of tokenism, constrained participation and limited influence. These limitations reflect deeper epistemic and relational inequities within mental health systems.

**Discussion:**

This commentary argues that the challenges of co‐design can be understood through the lens of epistemic injustice, where certain forms of knowledge, particularly lived experience, are systematically devalued. Drawing on restorative justice principles, the paper proposes a relational and ethical reframing of participation. Restorative approaches—centred on truth‐telling, acknowledgement and repair—offer a means to address the relational harms and power imbalances that undermine meaningful participation. Building on this, the Cycle of Equitable Codesign is introduced as a staged framework comprising: (1) acknowledgement and truth‐telling, (2) restoration and relational repair and (3) co‐design and collective future‐making. The model emphasises the importance of timing, power and relational conditions in shaping equitable participation.

**Conclusion:**

Repositioning co‐design within a restorative framework highlights the need to move beyond procedural inclusion towards ethically grounded and relationally just practice. Addressing epistemic injustice is essential for co‐design to achieve its transformative potential in mental health systems.

**Patient and Public Contribution:**

This commentary is informed by existing literature on co‐design and lived experience participation in mental health. It centres and reflects on the reported experiences of people with lived experience of mental health challenges. Although no direct formal patient or public involvement was undertaken in the development of this paper, it was informed following extensive informal communication with those who have a lived experience of mental health challenges and codesign participation over the prior 18 months.

## 1 Introduction

Mental health is a foundational determinant of overall wellbeing, shaping people's capacity to think, feel, relate, work and participate in community life. Despite decades of reform, mental health systems across many jurisdictions continue to struggle to respond effectively to the needs of the populations they serve. Alongside persistent service gaps and inequities, people with lived experience of mental health challenges frequently report feeling excluded, unheard or marginalised within the very systems intended to support them.

In response to these challenges, codesign has become increasingly prominent in mental health research, policy, and service development as a response to the limitations of traditional, often medical, professionally driven models of care. Drawing on participatory and experience‐based design approaches, the aim of co design is to position people with lived experience of mental health challenges (and carers), as active partners in the design, implementation and evaluation of services. Within mental health settings, co‐design has been shown to enhance service relevance, acceptability and engagement by ensuring that interventions reflect the priorities, values and contexts of those who use them. Further to this, there are strong signs that involving people with lived experience can improve the quality of mental health services and research outcomes [1, 2]. As such, codesign is increasingly seen as aligning with person‐centred frameworks, and is highly regarded as the preferred means of improving outcomes within the mental health system.

However, while the rhetoric of participation has expanded rapidly, practice has often tended to fall short [3]. For example, in contrast to the reported positive systemic impact, codesign participants regularly describe experiences of tokenism, constrained agendas and limited influence over outcomes. These challenges are not merely procedural failures but suggest deeper epistemic and relational dynamics within mental health systems.

This commentary argues that many of the reported limitations of codesign in mental health can be understood through the lens of epistemic injustice, the systematic devaluing of certain forms of knowledge and knowers [4]. It proposes that restorative approaches, particularly those drawn from restorative justice traditions, often seen within either the justice or education systems, offer a coherent ethical and practical foundation for addressing these injustices. Building on this argument, the commentary introduces ‘The Cycle of Equitable Codesign’, a staged, restorative framework designed to support more equitable, legitimate and ethically grounded codesign processes in mental health contexts.

### 1.1 Codesign to Combat Epistemic Injustice in Mental Health

Codesign refers to a range of participatory activities that bring together people, those who use services (consumers), carers, clinicians and other stakeholders to shape service design, delivery or evaluation [1]. Within mental health services, codesign aligns closely with person‐centred frameworks and is often presented as a holistic, and participatory means to enhance and improve models of care. In principle, it positions lived experience alongside professional (or clinical) expertise as a valued source of knowledge [2].

In practice, however, codesign frequently operates within systems characterised by entrenched power asymmetries. Traditionally, Western mental health systems have focused on educated, and sometime privileged, clinical, biomedical, knowledge considered as objective, rational and authoritative, while viewing the lived experience as the opposite experience, subjective, emotionally‐driven and deficit‐based. These viewpoints create hierarchies that shape what is considered as credible, actionable or legitimate knowledge in service settings.

The concept of epistemic injustice captures this complex dynamic. In mental health contexts, this may take the form of testimonial injustice, where the contributions of people with lived experience are discounted, reinterpreted, or overridden or hermeneutical injustice, where people lack shared conceptual resources to make sense of their experiences within dominant institutional frameworks [4].

Importantly, epistemic injustice is not an abstract or incidental concern. It is experienced materially by people who repeatedly argue their accounts are minimised, reframed, or, at worst, they from decision making. In codesign spaces, this can manifest when lived experience input is sought but subsequently marginalised, when agendas are pre‐determined (considered as ‘consultation’), or when participants are invited to share personal experiences without any authority to influence the possible outcomes [5]. Rather than redistributing this epistemic authority, these processes risk reproducing the very power differential they are seeking to address under the guise of ‘participation’.

### 1.2 Why Restorative Approaches Matter

Restorative approaches offer a way of responding to epistemic injustice that extends beyond procedural inclusion [6]. Originating in justice system contexts, restorative justice was developed as a response to harm, emphasising truth telling, accountability, repair and the restoration of relationships between those harmed, those who caused harm, and the broader community. Over time, restorative principles have been adapted across diverse settings, including education and healthcare, to support relational safety, learning and trust [7, 8]. Prior research examining challenges in co‐design within mental health settings has identified the need for ‘education and more open dialogue, such as a proactive restorative approach, to address stakeholder expectations and power imbalances’ [9]. This reinforces the relevance of restorative principles as a practical and ethical response to the relational challenges that emerge in participatory processes.

At their core, restorative approaches are concerned with both repair and reconnection. They seek to acknowledge harm, legitimise lived experiences of impact, and re‐establish relational and moral standing. Crucially, restorative processes do not presume consensus, forgiveness, or closure. Their ethical function is to create conditions under which dialogue and future collaboration can occur without coercion or erasure [8].

In healthcare settings, there have been limited uses of restorative approaches, however, such an approach has the potential to improve trust, psychological safety and organisational learning, as well as reductions in harm and cost [8]. Yet, the potential contribution of restorative approaches within participatory design and knowledge production remains underexplored. This is a significant omission given that codesign is fundamentally a communicative and relational practice, dependent on whose voices are heard, how meaning is negotiated and how decisions are justified.

A restorative lens highlights a critical but often overlooked issue in codesign, that of timing. Inviting people to collaborate on future solutions without first acknowledging past or ongoing harms may place the burden of reconciliation on those already marginalised. In such circumstances, participation becomes a demand for cooperation or consultation rather than a genuine partnership. Recognising this, restorative traditions emphasise that certain forms of participation are unethical or ineffective if initiated before acknowledgement and repair have occurred [6, 8].

### Staged Participation and the Ethics of Timing

Many critiques of codesign treat participation as a singular activity, overlooking the ethical significance of when and how different participatory purposes are invited. This commentary proposes staged participation as a means of responding to these critiques while retaining the transformative potential of codesign.

Staged participation recognises that participation is not neutral or universally appropriate. Different moments in reform, research or service improvement processes require distinct participatory aims, practices and power conditions. Drawing on restorative and justice‐oriented literature, this approach suggests that participation oriented towards future making should not occur before truth telling, acknowledgement and relational groundwork have been established [6].

**Figure 1:**
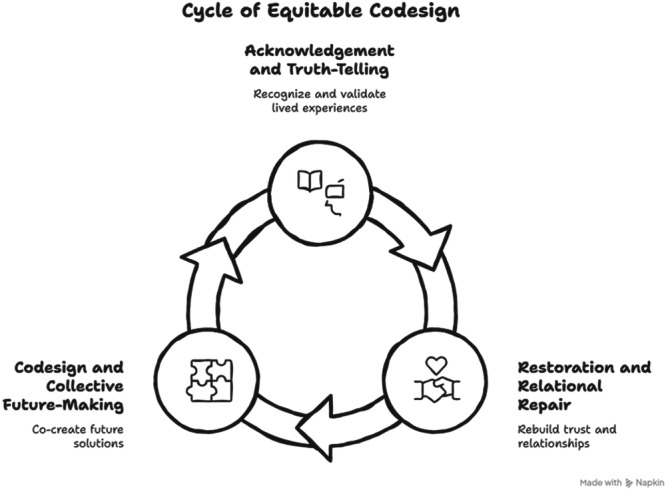
The Cycle of Equitable Codesign.

This insight forms the basis of ‘The Cycle of Equitable Codesign’, a restorative framework for mental health codesign that situates codesign within a broader ethical trajectory rather than treating it as a stand‐alone method (see Figure 1).

## 2 The Cycle of Equitable Codesign in Mental Health

*The Cycle of Equitable Codesign* is presented as a continuous, iterative framework comprising three interrelated stages: (1) Acknowledgement and truth telling, (2) restoration and relational repair and (3) codesign and collective future making. The cycle is not linear; rather, it assumes that power, trust and legitimacy require ongoing attention across the life of participatory work.

### Stage 1: Acknowledgement and Truth Telling (Pre‐Design)

The first stage centres on acknowledging harm and legitimising lived experience as authoritative knowledge about what has occurred. Its purpose is not problem solving or consensus building, but recognition, validation and epistemic repair. Appropriate participatory practices at this stage include testimony, storytelling, reflective dialogue, creative expression and formal submissions led by those most affected.

Crucially, silence, refusal, anger or ambivalence must be recognised as legitimate forms of participation choice. Where harm has been inflicted or denied at a systemic level, inviting immediate codesign risks re‐inscribing epistemic injustice by demanding collaboration without recognition. This stage establishes moral standing and begins to rebalance epistemic authority by publicly acknowledging lived experience as credible and consequential.

### 2.1 Stage 2: Restoration and Relational Repair

The second stage focuses on restoring trust, legitimacy and relational conditions for future engagement. Participation here is relational rather than generative, involving facilitated dialogue, shared exploration of impacts and clarification of responsibilities. Unlike the first stage, this phase may involve those who have caused or enabled harm, but participation must be carefully supported to ensure psychological safety [8].

Restorative principles such as equal concern, responsibility taking and attention to substantive, procedural and psychological justice are central at this stage. Restoration does not require agreement, forgiveness or closure. Its function is to enable participation to move from coerced or extractive engagement towards voluntary and ethically grounded collaboration through active listening.

### 2.2 Stage 3: Codesign and Collective Future Making

Only once acknowledgement and relational repair have occurred does codesign become ethically and practically viable. At this stage, participation shifts towards future making: co‐ideation, shared sense‐making, distributed leadership and collective decision‐making [1]. Codesign is more likely to be experienced as meaningful when it is grounded in recognised histories, repaired relationships and negotiated roles. Importantly, the risks highlighted in critiques of codesign such as tokenism, elite capture (where participation is dominated by more powerful or privileged voices) and epistemic exploitation can then be mitigated when power relations have already begun to shift [5].

Rather than asking participants to speak into predetermined agendas, this staged approach supports codesign as a space where the framing of problems and priorities can itself be reshaped.

### 2.3 Recognising Power as an Ongoing Practice

Central to the Cycle of Equitable Codesign is the explicit recognition of power. Power operates at multiple levels within mental health systems, including legislative and institutional mandates, professional hierarchies, funding arrangements and knowledge norms. Codesign that fails to interrogate these dynamics risks reproducing credibility deficits, even when participation is well‐intentioned.

Recognising power involves making visible how decisions are made, who holds authority, and which forms of knowledge are prioritised. It shifts the focus from whether people are invited to participate to whether they are meaningfully heard and able to influence outcomes. Importantly, this reframing locates responsibility within systems of silencing rather than within individuals who are positioned as lacking a voice.

### 2.4 Restorative Communication and Epistemic Repair

Restorative communication plays a critical role in supporting epistemically ‘just’ codesign. Such communication is explicit, non‐blaming and oriented towards understanding impact rather than allocating fault. Restorative questions invite reflection and perspective taking, while affective statements surface the relational and emotional consequences of decisions without personal accusation [8].

By structuring dialogue in this way, restorative communication supports psychological safety and shared understanding, particularly in contexts where trust has been eroded. It also creates pathways for in‐process repair when harm occurs, recognising that codesign itself can be a site of epistemic risk.

### 2.5 Implications for Mental Health Practice and Research

The proposed *Cycle of Equitable Codesign* reframes participation as a dynamic, context‐sensitive ethical practice rather than a single methodological choice. It responds to critiques of codesign not by abandoning participatory approaches, but by situating them within a broader restorative infrastructure. For practitioners, this framework invites reflection on the intent and timing of participation. For researchers, it highlights the need to attend to epistemic conditions alongside methodological rigour. By foregrounding power, timing and restoration, the model offers a pathway towards codesign that is not only inclusive in form, but equitable in substance. Future work within the mental health codesign space should be encouraged to test the model, or the possible variations, and report on the process and outcomes from the approach. Testing of the approach is key to moving forward within the codesign space.

### 2.6 Limitations

Restorative approaches are not immune to co‐option. By co‑option, this commentary refers to the risk that restorative language, tools or rituals may be adopted superficially within existing institutional structures without altering underlying power relations. In such cases, restorative processes can become procedural or symbolic, used to manage dissatisfaction, diffuse critique or signal organisational responsiveness, while decision‐making authority and epistemic control remain unchanged. When restoration is treated as a technique rather than an ethical orientation, there is a risk that dialogue substitutes for accountability, and participation is invited without a corresponding redistribution of power or resources. As with codesign itself, restorative approaches therefore require sustained institutional commitment, reflexivity and willingness to be shaped by critique. Without this, restorative practices may inadvertently reproduce the very epistemic and relational harms they seek to address, reinforcing legitimacy for systems rather than repairing trust for those who have been marginalised.

## 3 Conclusion

If codesign in mental health is to move beyond aspiration and rhetoric, it must confront the epistemic conditions that shape who is heard, whose knowledge counts, and how decisions are justified. Epistemic injustice is not peripheral to codesign; it is a central structural challenge that risks reproducing the very inequities participation seeks to dismantle.

This commentary has argued that restorative approaches provide a coherent and underutilised response to this challenge. By integrating acknowledgement, relational repair and staged participation, ‘The Cycle of Equitable Codesign’ reframes codesign as a relational and ethical practice grounded in justice rather than mere inclusion. In mental health systems, where voices have too often been marginalised or dismissed, such an approach is not optional, but essential.

## Author Contributions

The author confirms being the sole contributor of this work, including the conception, and the drafting and critical revision of the manuscript’ or similar which is standard for HEX.

## Funding

The author has nothing to report.

## Ethics Statement

The author has nothing to report.

## Consent

The author has nothing to report.

## Conflicts of Interest

The author declares no conflicts of interest.

## Data Availability

Data sharing is not applicable to this article as no datasets were generated or analysed during the current study.
